# Long-term incense use and the risk of end-stage renal disease among Chinese in Singapore: the Singapore Chinese health study

**DOI:** 10.1186/s12882-018-1186-9

**Published:** 2019-01-09

**Authors:** Ting-Ting Geng, Tazeen Hasan Jafar, Jian-Min Yuan, Woon-Puay Koh

**Affiliations:** 1 Saw Swee Hock School of Public Health, National University of Singapore, Singapore, Singapore; 2 Health Services and Systems Research, Duke-NUS Medical School, 8 College Road Level 4, Singapore, 169857 Singapore; 3 Department of Renal Medicine, Singapore General Hospital, Singapore, Singapore; 4 Division of Cancer Control and Population Sciences, UPMC Hillman Cancer Center, University of Pittsburgh, Pittsburgh, PA USA; 5 Department of Epidemiology, Graduate School of Public Health, University of Pittsburgh, Pittsburgh, PA USA

**Keywords:** End-stage renal disease, Incense, Prospective cohort study, Chinese

## Abstract

**Background:**

Experimental studies have shown that exposure to incense burning may have deleterious effects on kidney function and architecture. However, the association between chronic exposure to incense smoke and risk of end-stage renal disease (ESRD) has not been reported in epidemiologic studies.

**Methods:**

We investigated this association in the Singapore Chinese Health Study, a prospective population-based cohort of 63,257 Chinese men and women of 45–74 years of age in Singapore during recruitment from 1993 to 1998. Information on the practice of incense burning at home, diet, lifestyle and medical history was collected at baseline interviews. ESRD cases were identified through linkage with the nationwide Singapore Renal Registry through 2015. We used Cox proportional hazards regression analysis to estimate hazard ratio (HR) and 95% confidence interval (CI) of ESRD associated with domestic incense burning.

**Results:**

Among cohort participants, 76.9% were current incense users. After an average 17.5 years of follow-up, there were 1217 incident ESRD cases. Compared to never users, the multivariable-adjusted HR for ESRD risk was 1.05 (95% CI, 0.80 to 1.38) for former users and 1.26 (95% CI, 1.02 to1.57) for current users of incense. In analysis by daily or non-daily use and duration, the increased ESRD risk was observed in daily users who had used incense for > 20 years; HR was 1.25 (95% CI, 1.07 to 1.46). Conversely, the risk was not increased in those who did not use incense daily or who had used daily but for ≤20 years.

**Conclusions:**

Our findings demonstrate that long-term daily exposure to domestic incense burning could be associated with a higher risk of ESRD in the general population.

**Electronic supplementary material:**

The online version of this article (10.1186/s12882-018-1186-9) contains supplementary material, which is available to authorized users.

## Background

End-stage renal disease (ESRD), defined as kidney failure requiring dialysis or kidney transplantation, is a rising but poorly documented global public health threat associated with high morbidity and mortality rates, and with a significant economic burden borne by both individuals and health systems [1, 2]. The largest gaps of ESRD prevention and treatment were noted in low-income and middle-income countries, particularly in Asia [3, 4]. A cross-sectional study including China, India and Iran reported that only 6% of the general population were aware of their chronic kidney disease (CKD) status, and a substantial number of people did not receive treatments even though they knew they had CKD [5].

Incense burning at home is a traditional and common practice for ritual or religious purpose among the populations in Asian countries such as China [6], Singapore [7], Taiwan [8], and India [9], and also in the Arabian Gulf countries [10]. Incense burning generates a complex mixture of gases and particulate matter, which is known to be deleterious to human health [11, 12]. In fact, an in-vitro study has shown that the genotoxicity of particulate matter from incense burning could be higher than that from cigarette smoke [13]. Epidemiological studies suggest that incense use is associated with elevated blood pressure [6], and higher risks of cardiovascular disease morbidity and mortality [7, 14], upper respiratory tract cancers [15], and nasopharyngeal cancer [16].

Experimental studies have demonstrated that exposure to environmental air pollution may disturb renal hemodynamics, worsen renal oxidative stress, promote inflammation, and cause DNA damage in murine models [17, 18]. Recently, epidemiological studies suggest that exposure to particulate matter, nitrogen dioxide and carbon monoxide from outdoor or traffic-related air pollution is associated with increased risks of CKD and ESRD in the general population [19–22]. Characterization of emission from smoldering incense reveals that incense smoke contains the similar toxic particulate-bound chemicals as outdoor air pollutants [23–25], which suggests that exposure to incense burning may also have deleterious effects on CKD and progression to ESRD. In fact, deleterious effects of incense smoke exposure on kidney function and architecture in male albino rats have been documented in experimental studies [26]. However, no epidemiological study has examined the association between chronic incense exposure and renal function.

Singapore is the example of an Asian country where the prevalence of incense use is high. Furthermore, in Singapore, the incidence of ESRD has doubled over the past 20 years, and the country is currently ranked sixth in the world for incidence of treated ESRD in the United States Renal Data System (USRDS) Annual Data Report [27]. In this study, we investigated the association between domestic incense use and risk of ESRD among middle-aged or older Chinese in Singapore. We hypothesized that domestic use of incense could be associated with increased risk of ESRD in this population.

## Methods

### Study population

The Singapore Chinese Health Study is a population-based prospective cohort study, established between April 1993 and December 1998. The study recruited 63,257 Chinese (35,298 women and 27,959 men), aged 45–74 years, and residing in government-built housing estates, where 86% of the Singapore population resided at the time of recruitment. The recruitment was restricted to the two major dialect groups of Chinese in Singapore, the Hokkien, and Cantonese, who originated from the contiguous provinces of Fujian and Guangdong in the southern part of China, respectively [28]. All participants gave written informed consent. The study was approved by the Institutional Review Board of the National University of Singapore.

### Data collection

Trained interviewers did face-to-face interviews with participants at recruitment using a structured, scanner-readable questionnaire to collect information on demographics, height, weight, lifetime use of tobacco, alcohol intake, habitual physical activity, and self-reported medical history, including physician-diagnosed hypertension, coronary artery disease, stroke and diabetes. Usual dietary intake was assessed using a validated 165-item, semi-quantitative food frequency questionnaire.

For cigarette smoking, participants were categorized as never, former or current smokers based on their response to the following question, “Have you ever smoked at least one cigarette a day for one year or longer”. Participants were classified as “never-smokers” if they answered “no”, “former smokers” if they answered “yes, but I quit smoking”, and “current smokers” if they answered “yes, and I currently smoke” Ever smokers referred to former and current smokers.

### Assessment of incense exposure

We asked the participants whether their household ever burned incense (yes or no); if the answer was “yes,” we asked them whether they had burnt incense in the past one year. Those who had never burnt incense were classified as never users, and those who had used incense but not in the past one year were classified as former users. Non-users in our analysis included never users and former users. Among the current users who had used incense in the past one year, they were asked the number of years of incense use (10 years or less, 11–20, 21–30, 31–40, or 41 years or more), frequency of incense burning (daily, a few times per week, a few times per month, or a few times per year), placement of the main altar (kitchen, dining room, living room, other bedroom, or participant’s bedroom) and the intensity of burning (at all times, intermittently during the day, during the day only, or during the night only).

### Ascertainment of incident ESRD cases

We identified ESRD cases by linking the cohort database with the population-based Singapore Renal Registry, which has been shown to be comprehensive in the ESRD recording through multiple sources including laboratory records, hospital records, and listings of patients on dialysis [29]. Cases of ESRD were registered in the Renal Registry if they met at least one of the following criteria:1) estimated glomerular filtration rate (eGFR) < 15 ml/min per 1.73 m^2^, 2) serum creatinine level ≥ 880 μmol/L (10 mg/dl), 3) treatment with hemodialysis or peritoneal dialysis, or 4) had undergone kidney transplant. The first three criteria had to be persistent over 3 months to be qualified as a diagnosis for ESRD. Since 2010, the criteria have been revised to 1) serum creatinine ≥500 μmol/L, 2) eGFR < 15 ml/min/1.73 m^2^, or 3) if renal replacement therapy has been started [29, 30]. The linkage to identify ESRD cases among our SCHS participants was done through perfect matching of each participant’s unique National Identification Card Number used in our study as well as in the Renal Registry. As of Dec 31st 2015, only 57 subjects (< 0.1%) were lost to follow-up from our cohort due to migration or for other reasons, which suggests that emigration among the participants was negligible and that vital statistics were virtually complete.

### Statistical analysis

The current analysis included data from 63,147 participants after excluding 110 participants with ESRD diagnosed before enrollment into the cohort. We computed person-years from the date of recruitment to date of reported ESRD, loss to follow-up, death, or December 31st 2015, whichever occurred first. The differences in baseline characteristics by incense use status were examined using Student’s t-test for continuous variables and chi-squared test for categorical variables. Graphs for cumulative incidence of ESRD and death in non-users and current users were plotted using Nelson-Aalen cumulative hazard function. Differences in cumulative incidence of these two outcomes between these two groups were assessed by the log-rank test. We used multivariable Cox proportional hazards regression models to compute the HR and 95% CI for the association between incense use and risk of ESRD.

Model 1 was adjusted for the basic demographic factors: age (years), gender, dialect (Hokkien / Cantonese), year of baseline interview (1993–1995, 1996–1998), and education level (none, primary school, secondary school or higher). Besides these covariates, Model 2 further included lifestyle factors shown to affect risk of ESRD in our cohort or in the literature, i.e. body mass index (kg/m^2^), smoking status (never, former, current), physical activity (defined as any weekly moderate activity, vigorous activity or strenuous sports lasting ≥ ½ hour, yes or no), alcohol consumption (none, occasionally, weekly, daily), total energy intake (kcal/day), total protein consumption (quartiles), red meat consumption (quartiles), coffee consumption (none to < 1 cup/day, 1 cup/day, ≥ 2 cups/day), at least weekly ginseng intake (yes or no) and at least weekly medicinal soup intake (yes or no). For Model 3, we further adjusted for self-reported history of physician-diagnosed hypertension, stroke, coronary artery disease and diabetes (yes or no for each disease) to minimize the potential confounding effect of comorbidities on the observed association.

Previous studies have illustrated gender differences in risk of ESRD [31, 32]. Therefore we conducted stratified analysis by gender to look for differential association of incense with ESRD risk. In our previous study investigating incense use and cardiovascular disease mortality, smoking was a significant interaction factor in the analysis [7]. Hence, we also conducted stratified analysis by baseline smoking status (never and ever smokers) to examine for interaction between smoking and incense use in the association with ESRD risk. We used the likelihood ratio test to examine the interaction between smoking/gender and incense use by including an interaction term (product of smoking/gender and incense use) in the model. We conducted sensitivity analyses by excluding participants, including cases, with less than 4 years of follow-up to minimize the potential confounding effect of subclinical disease on the observed incense and ESRD association. We also repeated the analysis by setting December 31st 2009 as the censor date to see if the results were different using the old criteria for the definition of ESRD in the Registry.

All analyses were performed using Stata statistical software, release 14.0 (StataCorp LP, College Station, Texas). Statistical tests were two-sided, and *P* values of less than 0.05 were considered statistically significant.

## Results

In our study population, 76.9% among the participants were current users of domestic incense (77.4% among men and 76.4% among women). Only 13.0 and 10.1% participants were former users (defined as previous users but not in the past one year) and never users, respectively. Among the 48,545 current users, most had used it daily for over 20 years (90.0%). Vast majority of subjects placed their altars where incense was burnt in the living room (91.9%), while the remaining subjects placed their altars either in the kitchen (6.5%), dining room (1.2%) or bedroom (0.4%). Most current users burnt incense intermittently during the day (80.8%), while the remainder burnt incense throughout the day (11.0%), throughout the night (4.7%) or at all times (3.5%).

As shown in Table 1, compared with non-users, which included never and former users, current users were more likely to be Hokkien for dialect group, current smokers (21.2% versus 13.9%), coffee drinkers of ≥2 cups per day (35.6% versus 29.7%) and less educated (all *Ps* < 0.001). Current users were less likely to be physically active (31.5% versus 37.5%), and less likely to consume medicinal soup and ginseng (all *Ps* < 0.004). We also noted that current users had a slightly lower prevalence of coronary heart disease (4.0% versus 4.5%, *P* = 0.01) but a marginally higher prevalence of diabetes (9.2% versus 8.2%, *P* < 0.001). Results on the associations of these aforementioned factors and other covariates with the risk of ESRD could be found in Additional file 1: Table S1. Briefly, age, gender, BMI, dialect, education level, smoking, alcohol consumption, physical activity, coffee consumption, ginseng intake, red meat intake, and history of diabetes, hypertension and coronary heart disease were found to be associated with the risk of ESRD and included as covariates in the final Model 3.

**Table 1:** Baseline characteristics of participants by current use of incense in the Singapore Chinese Health Study (1993–1998) (*n* = 63,147)

Characteristic	Current users (*n* = 48,545)	Non-users (never and former users) (*n* = 14,602)	*P*-value^a^
Age at baseline (years)	56.5 ± 8.0	56.4 ± 8.2	0.02
BMI (kg/m^2^)	23.2 ± 3.3	22.9 ± 3.2	< 0.001
Gender			0.002
Men	21,607 (44.5)	6289 (43.1)	
Women	26,938 (55.5)	8313 (56.9)	
Dialect			< 0.001
Cantonese	21,455 (44.2)	7777 (53.3)	
Hokkien	27,090 (55.8)	6825 (46.7)	
Education			< 0.001
No formal education	14,977 (30.9)	2325 (15.9)	
Primary school (1–6 years)	22,948 (47.3)	5047 (34.6)	
≥ Secondary school	10,620 (21.9)	7230 (49.5)	
Cigarette smoking			< 0.001
Never	32,921 (67.8)	10,925 (74.8)	
Former	5333 (11.0)	1643 (11.3)	
Current	10,291 (21.2)	2034 (13.9)	
Alcohol consumption			0.01
None/monthly	42,851 (88.3)	12,986 (88.9)	
Weekly	3939 (8.1)	1166 (8.0)	
Daily	1755 (3.6)	450 (3.1)	
Physical activity^b^	15,286 (31.5)	5478 (37.5)	< 0.001
Coffee consumption			< 0.001
None to < 1 cup/day	13,771 (28.4)	5007 (34.3)	
1 cup/day	17,495 (36.0)	5262 (36.0)	
≥ 2 cups/day	17,279 (35.6)	4333 (29.7)	
Ginseng intake (at least weekly)	1226 (2.5)	432 (3.0)	0.004
Medicinal soup intake (at least weekly)	4656 (9.6)	1690 (11.6)	< 0.001
Total protein intake (g/day)	59.0 ± 9.9	59.7 ± 10.2	< 0.001
Red meat intake (g/day)	30.8 ± 18.8	29.6 ± 18.4	< 0.001
History of disease			
Diabetes mellitus	4468 (9.2)	1203 (8.2)	< 0.001
Hypertension	11,426 (23.5)	3546 (24.3)	0.1
Coronary heart disease	1937 (4.0)	650 (4.5)	0.01
Stroke	706 (1.5)	240 (1.6)	0.1

^a^ *P* values based on chi-square test for categorical variables and *t* test for continuous variables

^b^Physical activity defined as having any weekly moderate activity, vigorous activity or strenuous sports lasting at least 30 min

**Fig. 1:**
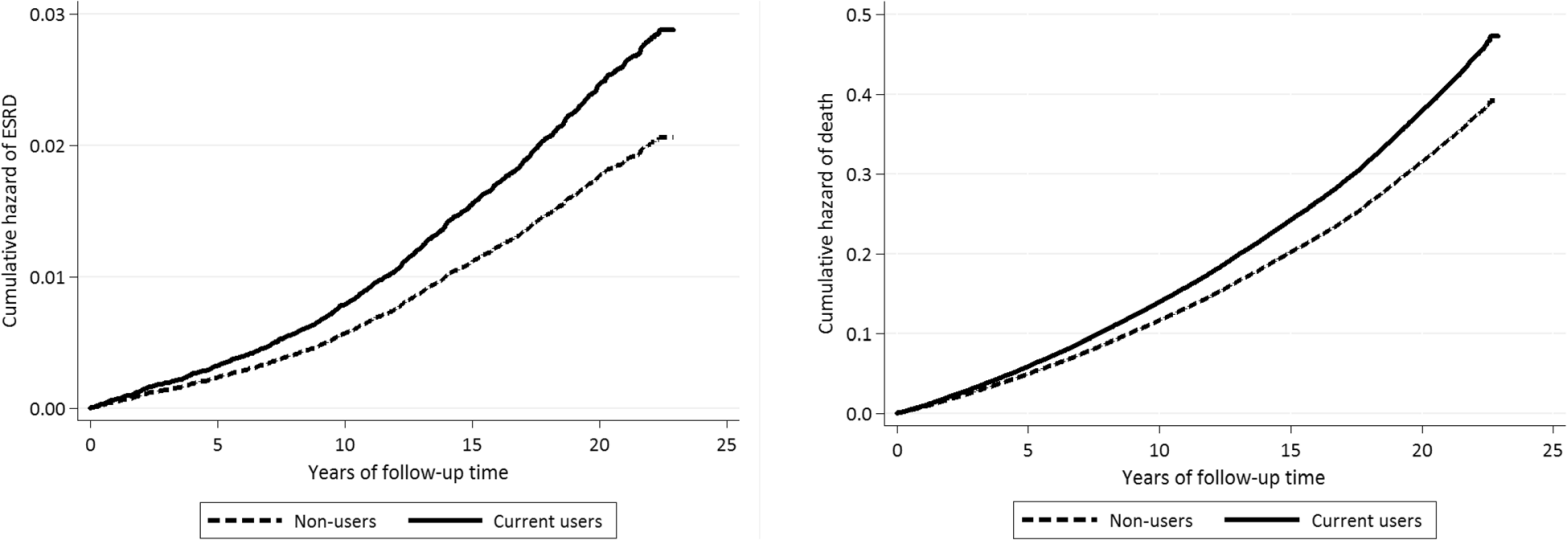
Cumulative incidence of ESRD and death by incense use status (non-users versus current users)

After a mean ± SD follow-up of 17.5 ± 5.5 years among 63,147 participants, we documented 1217 incident ESRD cases and 19,810 deaths. As shown in Fig. 1, the cumulative incidences for ESRD and total mortality were both higher among current users than non-users (both *Ps* for log-rank test < 0.001).

Exposure to incense burning was positively associated with risk of ESRD in a model that adjusted for basic demographic characteristics (Model 1); compared to never users, current users had a hazard ratio (HR) of 1.34 [95% confidence interval (95% CI), 1.08 to 1.66]. The HR was 1.32 (95% CI, 1.07 to 1.64) after adjusting for other dietary and lifestyle risk factors (Model 2), and further attenuated to 1.26 (95% CI, 1.02 to 1.57) after additional adjustment for comorbidity factors (Model 3). Compared with never users, the HRs for the former users was 1.05 (95% CI, 0.80 to 1.38) after adjusting for all potential confounders. Compared with non-users, current users had a statistically significant 23% higher risk of developing ESRD (HR, 1.23; 95% CI, 1.05 to 1.43) after adjustment for all potential confounders (Table 2). We also created categories that incorporated information about daily usage and years of incense using. Compared with non-users, current users that did not use incense daily or daily users for ≤20 years did not have increased risk of ESRD. Conversely, the risk estimates were similar in both groups of daily users that had used incense for 21–40 years (HR, 1.33; 95% CI, 1.06 to 1.67) or for more than 40 years (HR, 1.24; 95% CI, 1.06 to 1.45), with overlap of the two 95% CIs. Hence, we combined them into a single category of daily users with more than 20 years of incense burning, and they had a 25% (HR, 1.25; 95% CI, 1.07 to 1.46) higher risk compared to non-users (*P*-trend = 0.003). The increased risk of ESRD associated with current incense users was more apparent in women than men, and higher in never smokers than smokers, but these differences were not statistically significant (*P* heterogeneity ≥0.07) (Table 3).

**Table 2:** Hazard ratios (95% CI) for risk of ESRD according to incense use (*n* = 63,147)

Exposure	Participants (n)/Person-years	ESRD			
		Cases	Model 1	Model 2	Model 3
Status of incense use					
Never users	6365/114,369	95	1.00 (reference)	1.00 (reference)	1.00 (reference)
Former users	8237/144,437	124	1.10 (0.84–1.44)	1.11 (0.84–1.45)	1.05 (0.80–1.38)
Current users	48,545/845,858	998	1.34 (1.08–1.66)	1.32 (1.07–1.64)	1.26 (1.02–1.57)
Non-users (never and former users)	14,602/248,167	219	1.00 (reference)	1.00 (reference)	1.00 (reference)
Current users	48,545/812,982	998	1.27 (1.09–1.48)	1.25 (1.08–1.46)	1.23 (1.05–1.43)
Frequency and duration of incense use					
Non-users (never and former users)	14,602/258,806	219	1.00 (reference)	1.00 (reference)	1.00 (reference)
Current, less than daily users	3570/62,603	67	1.12 (0.85–1.48)	1.09 (0.83–1.44)	1.06 (0.81–1.40)
Current, daily users for ≤20 years	1310/24,268	17	0.93 (0.57–1.52)	0.95 (0.58–1.55)	0.98 (0.60–1.61)
Current, daily users for 21–40 years	5857/108,441	123	1.33 (1.06–1.67)	1.35 (1.07–1.70)	1.33 (1.06–1.67)
Current, daily users for > 40 years	37,808/650,547	791	1.29 (1.10–1.51)	1.27 (1.08–1.48)	1.24 (1.06–1.45)
*P* for trend			0.001	0.002	0.004

Model 1: adjusted for age at recruitment (years), gender, dialect (Cantonese, Hokkien), education level (no formal education, primary school, ≥ secondary school) and year of interview (1993–1995, 1996–1998)

Model 2: model 1 plus body mass index (kg/m^2^), physical activity (any weekly moderate activity, vigorous activity or strenuous sports lasting at least 30 min: yes or no), smoking status (never, former, current smokers), alcohol use (none, monthly, weekly, daily), total energy intake (kcal/day), total protein intake (gram/day, quartiles), red meat consumption (gram/day, quartiles), coffee consumption (None to < 1 cup/day, 1 cup/day, ≥ 2 cups/day), weekly ginseng intake (yes or no) and weekly medicinal soup intake (yes or no)

Model 3: model 2 plus self-reported history of physician-diagnosed hypertension, diabetes, coronary heart disease and stroke (yes or no)

**Table 3:** Hazard ratios (95% CI) for risk of ESRD according to incense use, stratified by gender and smoking (*n* = 63,147)

Exposure	Participants (n)		ESRD		*P* for interaction
		Person-years	Cases	HR (95%CI)	
Stratified by gender^a^					0.07
Men					
Non-users^b^	6289	106,431	115	1.00 (reference)	
Current users	21,607	357,800	435	1.12 (0.91–1.39)	
Women					
Non-users	8313	152,375	104	1.00 (reference)	
Current users	26,938	488,059	563	1.35 (1.08–1.67)	
Stratified by smoking status^c^					0.45
Never-smokers					
Non-users	10,925	200,202	150	1.00 (reference)	
Current users	32,921	599,278	671	1.26 (1.05–1.52)	
Ever smokers					
Non-users	3677	58,603	69	1.00 (reference)	
Current users	15,624	246,580	327	1.15 (0.88–1.50)	

^a^The estimates were generated using Cox proportional hazards models, with adjustment for age at recruitment, dialect, education level, year of interview, body mass index, physical activity, smoking status, alcohol use, total energy intake, total protein intake, red meat consumption, coffee consumption, weekly ginseng intake, weekly medicinal soup intake and self-reported history of physician-diagnosed hypertension, diabetes, coronary heart disease and stroke

^b^Non-users were defined as never users and former users

^c^The estimates were generated using Cox proportional hazards models, with adjustment for age at recruitment, gender, dialect, education level, year of interview, body mass index, physical activity, alcohol use, total energy intake, total protein intake, red meat consumption, coffee consumption, weekly ginseng intake, weekly medicinal soup intake and self-reported history of physician-diagnosed hypertension, diabetes, coronary heart disease and stroke

We conducted sensitivity analysis that excluded ESRD cases (*n* = 1066) and participants with less than 4 years of follow-up and the results were virtually unchanged; compared with non-users, current and daily users of incense for more than 20 years had 23% increased risk of ESRD (HR, 1.23; 95% CI: 1.05 to 1.45). We also repeated the analysis using the old criteria of ESRD definition in the Registry by setting December 31st 2009 as the censor date, and the results remained the same; compared with non-users, current and daily users of incense for more than 20 years had 27% higher risk of ESRD (HR, 1.27; 95% CI: 1.04 to 1.55).

## Discussion

Our data from this cohort of middle-aged and older Chinese in Singapore showed that daily exposure to domestic use of incense for more than 20 years was associated with an increased risk of developing ESRD.

To our best knowledge, this is the first study to provide epidemiological evidence of a relation between domestic incense burning and risk of ESRD. Incense burning for ritual, religious or meditation purposes is a very common practice in many countries and regions. A study in the United Arab Emirates reported that 86.0% of the participants were exposed to incense smoke at least once a week and 44.0% of them were exposed daily [33]. Studies in China (76.1%), India (78.0%) and Thailand (67.4%) also reported high proportions of current incense users in their respective study populations [6, 14, 34]. In our study in Singapore, the prevalence of current incense use was 76.9%. Thus, our findings are of tremendous global health relevance.

Incense burning could generate considerable concentrations of particulate matter and a multitude of harmful chemicals that are similar to the traffic-related air pollutants. Characterization of smoke from incense burning has shown that the gaseous phase of incense emission consists of carbon dioxide, carbon monoxide, sulfur dioxide, formaldehyde, nitrogen dioxide, polycyclic aromatic compounds (PAHs), volatile organic compounds (VOCs), as well as particulate matters defined as PM_10_ (diameter ≤ 10 um), fine (diameter ≤ 2.5um) and ultrafine (diameter ≤ 0.1um) particles [23, 24, 35, 36]. In fact, the domestic concentration of fine particulate matter of incense burning may far exceed the outdoor standards specified by the World Health Organization [37].

Epidemiologic studies have shown that long-term exposure to incense burning in the home environment is associated with an increased risk of cardiovascular mortality. One cross-sectional study showed that compared with non-users, daily users of incense had a higher prevalence of increased carotid intima-media thickness [14]. In our own study using data from the Singapore Chinese Health Study, we have also reported that daily exposure to incense burning at home for over 20 years was associated with a higher risk of cardiovascular mortality compared to non-users.^2^ Although no previous epidemiological study has investigated the association between incense use and risk of ESRD, several studies have investigated the associations between outdoor pollution, such as traffic-related air pollutions, and deterioration of eGFR, risk of CKD and progression to ESRD. Two cross-sectional studies conducted in Taiwan using land-use regression modelling showed that exposure to traffic-related air pollutants, including PM_10_, PMcoarse (PM_2.5–10_), PM_2.5_ and NO_2,_ was inversely associated with eGFR and positively associated with the prevalence of CKD among adults over 30 years of age, and especially among elderly residents over 65 years of age [20, 21]. A recent large cohort study in US comprising 2,482,737 participants, including 357,600 CKD cases and 31,790 ESRD cases, found that a 10-ug/m^3^ increase in particulate matter (PM_2.5_) concentration was associated with increased risk of CKD and progression to ESRD [19]. Hence, our finding is not unexpected, given the similarity in the composition of pollutants in incense smoke and traffic-related emission.

The mechanism underlying the link between incense burning and ESRD has not been fully elucidated. A recent study in an animal model suggested that adverse modulating effects of incense smoke on kidney functions and architecture in rats could be mediated through augmented oxidative stress and inflammation. In this study, rats exposed to incense smoke showed deterioration in normal physiological functions with a substantial increase in serum creatinine, uric acid and blood urea nitrogen, and significant ultrastructural changes in kidney tissues. This was accompanied by a corresponding increase in these animals of inflammatory markers such as tumor necrosis factor-alpha and interleukin-4 levels, an increase in oxidative stress indicated by a significant increase in tissue malondialdehyde and tissue gene expression of both CYP1A1 and CYP1A2, and a decline in tissue reduced glutathione and catalase activity [26].

In our study, we found that the positive association between incense use and ESRD seemed to be stronger in never smokers; nevertheless, the interaction between incense smoke and cigarette smoking on the risk of ESRD was not statistically significant. Cigarette smoking has been well been recognized as an oxidative stressor that induces the accumulation of reactive oxygen species and overexpression of NADPH oxidase 4, which could in turn lead to renal vasoconstriction, sodium retention and worsen glomerular injury [38]. We have previously shown that ever smokers (former and current smokers) had a higher risk of ESRD compared to never smokers [39]. Since both incense smoke and cigarette smoke may affect renal function via the same biological pathway, it would not be surprising for the effect of incense smoke to be more prominent in never smokers compared to ever smokers. Based on the existing evidence, we inferred that the detrimental effects of incense on kidney function could have lesser effect for smokers who were already exposed to the strong and harmful substances from cigarette smoking, when compared to non-smokers. We also found a more pronounced effect in women than men. This stronger effect in women compared to men was consistent with a study which showed a stronger positive association of particulate matter exposure with CKD in women [20]. It is possible that women are more likely to be exposed to domestic incense burning since women usually spend more time at home and are more likely to be engaged in ritual or religious practice than men [16]. Furthermore, women were more likely to be never smokers, and in our study population, the proportion of never smokers in women was three-fold the proportion in men.

The strengths of this study include its population-based prospective design, large sample size, detailed information on lifestyle factors that are established or potential risk factors of ESRD, the objective assessment of ESRD endpoints and the virtual completeness of follow-up by linkage with the national Singapore Renal Registry, which has strict definitions of ESRD that follow international guidelines [29]. It is noteworthy that two characteristics of our population make this study unique for the evaluation of the association between incense use and ESRD. First, the prevalence rate of incense use was high, and the majority of the current users had used incense for over 20 years. Second, Singapore is a tropical island country with relatively low levels of outdoor air pollution [40] and scarce use of solid fuel [41], which makes effects from other sources of air pollution negligible.

Several limitations should be acknowledged. First, we only measured the incense burning at recruitment. However, given that the majority of exposed participants had reported long-term daily exposures, and that the incense burning is related to religious or ritual practices, the probability of stopping the habit of incense use would be low. Supporting this hypothesis, in our study, only about 13% were former users. In addition, measurement error of self-report and one-time assessment of incense use may lead to non-differential misclassification that could attenuate the true association between incense use and risk of ESRD. Second, we did not collect the information on the types of incense used. However, Lee et al. assessed ten types of commonly used incense in Hong Kong and found that the particulate matter and harmful chemicals emitted from incense significantly exceeded the Recommended Indoor Air Quality in Hong Kong, regardless of the types of incense and even in the ones that claimed to be “environmental friendly” [24]. Third, we did not measure the specific characteristics and concentration of particulate matter emitted by incense burning in each household, and we did not collect the information on ventilation practices that may influence the concentration of indoor air pollutants, such as opening the windows or closing the interior doors while incense was burning. Fourth, we did not collect information on the duration of quitting the use of incense from former users to study the effect of cessation. Fifth, we did not measure any lipid biomarkers at baseline, and hence, we could not adjust for dyslipidemia as a potential confounder. Furthermore, we did not measure kidney function at recruitment and hence, we were unable to establish the temporal relationship between incense use and deteriorating renal function with certainty. However, we conducted sensitivity analysis that excluded participants with short follow-up time of 4 years or less in our attempt to overcome the potential confounding effect of subclinical renal impairment in cases prior to ESRD diagnosis, and the results were consistent. Finally, this study is limited by its observational design, and we cannot completely exclude the possibility that residual confounding may still affect the association between incense use and ESRD risk.

## Conclusion

Our study provides epidemiological evidence that long-term exposure to domestic incense smoke may contribute to the risk of ESRD in the general populations. We acknowledge the lack of information on kidney function at baseline as a limitation in our study, and recommend that the findings be corroborated by future studies that can demonstrate the deterioration in kidney function with time in incense users. Given the worldwide prevalence of incense burning, our finding has substantial public health implications. We advocate implementing strategies to reduce exposure to the emissions from domestic incense and educating the public about the importance of improving ventilation with the use of incense.

## Additional file

Additional file 1: **Table S1** Associations between baseline characteristics (covariates) and risk of ESRD. (DOC 66 kb)

## Abbreviations

BMI: Body mass index
CI: Confidence interval
CKD: Chronic kidney disease
eGFR: Estimated glomerular filtration rate
ESRD: End-stage renal disease
HR: Hazard ratio
SD: Standard deviation

## References

[1] Mills KT, Xu Y, Zhang W, Bundy JD, Chen CS, Kelly TN, Chen J, He J (2015). A systematic analysis of worldwide population-based data on the global burden of chronic kidney disease in 2010. Kidney Int.

[2] El Nahas M (2005). The global challenge of chronic kidney disease. Kidney Int.

[3] Liyanage T, Ninomiya T, Jha V, Neal B, Patrice HM, Okpechi I, Zhao MH, Lv J, Garg AX, Knight J (2015). Worldwide access to treatment for end-stage kidney disease: a systematic review. Lancet.

[4] Dare AJ, Fu SH, Patra J, Rodriguez PS, Thakur JS, Jha P (2017). Renal failure deaths and their risk factors in India 2001-13: nationally representative estimates from the million death study. Lancet Glob Health.

[5] Ene-Iordache B, Perico N, Bikbov B, Carminati S, Remuzzi A, Perna A, Islam N, Bravo RF, Aleckovic-Halilovic M, Zou H (2016). Chronic kidney disease and cardiovascular risk in six regions of the world (ISN-KDDC): a cross-sectional study. Lancet Glob Health.

[6] Song X, Ma W, Xu X, et al. The association of domestic incense burning with hypertension and blood pressure in Guangdong, China. Int J Environ Res Public Health. 2017;14(7). 10.3390/ijerph14070788 PMC5551226 28708101

[7] Pan A, Clark ML, Ang LW, Yu MC, Yuan JM, Koh WP (2014). Incense use and cardiovascular mortality among Chinese in Singapore: the Singapore Chinese health study. Environ Health Perspect.

[8] Chen YC, Ho WC, Yu YH (2017). Adolescent lung function associated with incense burning and other environmental exposures at home. Indoor Air.

[9] Dewangan S, Chakrabarty R, Zielinska B, Pervez S (2013). Emission of volatile organic compounds from religious and ritual activities in India. Environ Monit Assess.

[10] Cohen R, Sexton KG, Yeatts KB (2013). Hazard assessment of United Arab Emirates (UAE) incense smoke. Sci Total Environ.

[11] Bootdee S, Chantara S, Prapamontol T (2016). Determination of PM2.5 and polycyclic aromatic hydrocarbons from incense burning emission at shrine for health risk assessment. Atmos Pollut Res.

[12] Lui KH, Bandowe BAM, Ho KF, Ho SSH, Chuang H-C, Chuang K-J, Cao J-J, Lee SC, Hu D (2016). Characterization of chemical components and bioreactivity of fine particulate matter (PM2.5) during incense burning. Environ Pollut.

[13] Zhou R, An Q, Pan XW, Yang B, Hu J, Wang YH (2015). Higher cytotoxicity and genotoxicity of burning incense than cigarette. Environ Chem Lett.

[14] Kammoolkon R, Taneepanichskul N, Pitaknoppakul N, Lertmaharit S, Lohsoonthorn V (2018). Incense smoke and increasing carotid intima media thickness: a cross-sectional study of the Thai-Vietnamese community. Asia Pac J Public Health.

[15] Friborg JT, Yuan JM, Wang R, Koh WP, Lee HP, Yu MC (2008). Incense use and respiratory tract carcinomas: a prospective cohort study. Cancer.

[16] Xie SH, Yu IT, Tse LA, Au JS, Wang F, Lau JS, Zhang B (2014). Domestic incense burning and nasopharyngeal carcinoma: a case-control study in Hong Kong Chinese. Environ Mol Mutagen.

[17] Nemmar A, Al-Salam S, Zia S, Yasin J, Al Husseni I, Ali BH (2010). Diesel exhaust particles in the lung aggravate experimental acute renal failure. Toxicol Sci.

[18] Al Suleimani YM, Al Mahruqi AS, Al Za'abi M, Shalaby A, Ashique M, Nemmar A, Ali BH (2017). Effect of diesel exhaust particles on renal vascular responses in rats with chronic kidney disease. Environ Toxicol.

[19] Bowe B, Xie Y, Li T, Yan Y, Xian H, Al-Aly Z (2018). Particulate matter air pollution and the risk of incident CKD and progression to ESRD. J Am Soc Nephrol.

[20] Yang YR, Chen YM, Chen SY, Chan CC (2017). Associations between long-term particulate matter exposure and adult renal function in the Taipei Metropolis. Environ Health Perspect.

[21] Chen SY, Chu DC, Lee JH, Yang YR, Chan CC (2018). Traffic-related air pollution associated with chronic kidney disease among elderly residents in Taipei City. Environ Pollut.

[22] Bowe B, Xie Y, Li T, Yan Y, Xian H, Al-Aly Z (2017). Associations of ambient coarse particulate matter, nitrogen dioxide, and carbon monoxide with the risk of kidney disease: a cohort study. The Lancet Planetary Health.

[23] Ji X, Le Bihan O, Ramalho O, Mandin C, D'Anna B, Martinon L, Nicolas M, Bard D, Pairon JC (2010). Characterization of particles emitted by incense burning in an experimental house. Indoor Air.

[24] Lee S-C, Wang B (2004). Characteristics of emissions of air pollutants from burning of incense in a large environmental chamber. Atmos Environ.

[25] Wang B, Lee SC, Ho KF, Kang YM (2007). Characteristics of emissions of air pollutants from burning of incense in temples, Hong Kong. Sci Total Environ.

[26] Hussain T, Al-Attas OS, Alrokayan SA, Ahmed M, Al-Daghri NM, Al-Ameri S, Pervez S, Dewangan S, Mohammed A, Gambhir D, et al. Deleterious effects of incense smoke exposure on kidney function and architecture in male albino rats. Inhal Toxicol. 2016;28(8):364–73. 10.1080/08958378.2016.1179372 27180632

[27] 2017 USRDS annual data report: Epidemiology of kidney disease in the United States. https://www.usrds.org/adr.aspx. Accessed 10 2018.

[28] Hankin JH, Stram DO, Arakawa K, Park S, Low SH, Lee HP, Yu MC (2001). Singapore Chinese health study: development, validation, and calibration of the quantitative food frequency questionnaire. Nutr Cancer.

[29] Singapore Renal Registry Annual Registry Report 1999-2013 (Preliminary). https://www.nrdo.gov.sg/docs/librariesprovider3/Publications---Kidney-Failure/singapore-renal-registry-annual-registry-report-1999-2013-preliminary.pdf?sfvrsn=0. Accessed 20 Mar 2018.

[30] Singapore Renal Registry Annual Report 2016. https://www.nrdo.gov.sg/docs/librariesprovider3/default-document-library/singapore-renal-registry-annual-report-2015.pdf?sfvrsn=0. Accessed 20 Mar 2018.

[31] Eriksen BO, Ingebretsen OC (2006). The progression of chronic kidney disease: a 10-year population-based study of the effects of gender and age. Kidney Int.

[32] Halbesma N, Brantsma AH, Bakker SJ, Jansen DF, Stolk RP, De Zeeuw D, De Jong PE, Gansevoort RT (2008). Gender differences in predictors of the decline of renal function in the general population. Kidney Int.

[33] Yeatts KB, El-Sadig M, Leith D, Kalsbeek W, Al-Maskari F, Couper D, Funk WE, Zoubeidi T, Chan RL, Trent CB (2012). Indoor air pollutants and health in the United Arab Emirates. Environ Health Perspect.

[34] Elf JL, Kinikar A, Khadse S, Mave V, Suryavanshi N, Gupte N, Kulkarni V, Patekar S, Raichur P, Breysse PN et al. Sources of household air pollution and their association with fine particulate matter in lowincome urban homes in India. J Expo Sci Environ Epidemiol 2018;28(4):400-10. 10.1038/s41370-018-0024-2 PMC6013356 29789668

[35] Yang TT, Lin TS, Chang M (2007). Characteristics of emissions of volatile organic compounds from smoldering incense. Bull Environ Contam Toxicol.

[36] Yang TT, Lin TS, Wu JJ, Jhuang FJ (2012). Characteristics of polycyclic aromatic hydrocarbon emissions of particles of various sizes from smoldering incense. Bull Environ Contam Toxicol.

[37] Ho SS, Yu JZ (2002). Concentrations of formaldehyde and other carbonyls in environments affected by incense burning. J Environ Monit.

[38] Hua P, Feng W, Ji S, Raij L, Jaimes EA (2010). Nicotine worsens the severity of nephropathy in diabetic mice: implications for the progression of kidney disease in smokers. Am J Physiol Renal Physiol.

[39] Jin A, Koh WP, Chow KY, Yuan JM, Jafar TH (2013). Smoking and risk of kidney failure in the Singapore Chinese health study. PLoS One.

[40] Velasco E, Roth M (2012). Review of Singapore's air quality and greenhouse gas emissions: current situation and opportunities. J Air Waste Manag Assoc.

[41] Bruce N, Rehfuess E, Mehta S, Hutton G, Smith K: Indoor Air Pollution. In: Disease Control Priorities in Developing Countries. Edited by Jamison DT, Breman JG, Measham AR, Alleyne G, Claeson M, Evans DB, Jha P, Mills A, Musgrove P. Washington (DC): World Bank; 2006. 21250309

