# Supplementary material for: Long-term incense use and the risk of end-stage renal disease among Chinese in Singapore: the Singapore Chinese health study
Source: BMC Nephrol. 2019 Jan 9;20:9. doi: 10.1186/s12882-018-1186-9 (PMC6325774; doi:10.1186/s12882-018-1186-9)
Supplement: Supplementary file 1 — Table S1 Associations between baseline characteristics (covariates) and risk of ESRD. (DOC 66 kb) [file 12882_2018_1186_MOESM1_ESM.doc]

Additional file 1: Table S1. Associations between baseline characteristics (covariates) and risk of ESRD

| Characteristics | Case | Person-year | HR (95% CI)^a^ |
| --- | --- | --- | --- |
| Age at baseline (years) | 1,217 | 1,104,665 | 1.02 (1.01-1.03) |
| BMI (kg/m^2^) | 1,217 | 1,104,665 | 1.03 (1.02-1.05) |
| Gender |  |  |  |
| Men | 550 | 464,231 | 1.00 (reference) |
| Women | 667 | 640,434 | 0.71 (0.61-0.82) |
| Dialect |  |  |  |
| Cantonese | 480 | 508,804 | 1.00 (reference) |
| Hokkien | 737 | 595,681 | 1.23 (1.09-1.39) |
| Year of interview |  |  |  |
| 1993-1995 | 876 | 765,611 | 1.00 (reference) |
| 1996-1998 | 341 | 339,054 | 0.93 (0.81-1.07) |
| Education |  |  |  |
| No formal education | 447 | 291,010 | 1.00 (reference) |
| Primary school (1-6 years) | 509 | 487,974 | 0.76 (0.66-0.87) |
| ≥ Secondary school | 261 | 325,681 | 0.71 (0.59-0.85) |
| Cigarette smoking |  |  |  |
| Never | 821 | 799,481 | 1.00 (reference) |
| Former | 176 | 110,051 | 1.17 (0.98-1.41) |
| Current | 220 | 195,133 | 1.23 (1.04-1.46) |
| Alcohol consumption |  |  |  |
| None/monthly | 1,111 | 978,296 | 1.00 (reference) |
| Weekly | 65 | 90,493 | 0.77 (0.59-0.99) |
| Daily | 41 | 35,876 | 1.25 (0.91-1.73) |
| Physical activity lasting at least 30 minutes per week | | | |
| No | 871 | 733,192 | 1.00 (reference) |
| Yes | 346 | 371,474 | 0.81 (0.71-0.92) |
| Coffee consumption |  |  |  |
| None to < 1 cup/day | 421 | 324,823 | 1.00 (reference) |
| 1 cup/day | 441 | 398,969 | 0.89 (0.78-1.02) |
| ≥ 2 cups/day | 355 | 380,874 | 0.81 (0.70-0.94) |
| Ginseng intake |  |  |  |
| No | 1,180 | 1,076,762 | 1.00 (reference) |
| Yes | 37 | 27,903 | 1.77 (1.00-3.13) |
| Medicinal soup intake |  |  |  |
| No | 1,101 | 992,480 | 1.00 (reference) |
| Yes | 116 | 112,185 | 1.04 (0.85-1.27) |
| Red meat intake (g/day, in quartile) | |  |  |
| Q1 | 256 | 278,518 | 1.00 (reference) |
| Q2 | 277 | 277,116 | 0.99 (0.83-1.18) |
| Q3 | 351 | 274,652 | 1.27 (1.06-1.51) |
| Q4 | 333 | 274,379 | 1.26 (1.05-1.51) |
| Total protein intake (g/day, in quartile) | |  |  |
| Q1 | 240 | 273,316 | 1.00 (reference) |
| Q2 | 321 | 274,918 | 1.15 (0.96-1.37) |
| Q3 | 319 | 277,498 | 1.07 (0.89-1.29) |
| Q4 | 337 | 278,934 | 1.02 (0.85-1.24) |
| Diabetes mellitus |  |  |  |
| No | 756 | 1,025,504 | 1.00 (reference) |
| Yes | 461 | 79,161 | 5.44 (4.80-6.16) |
| Hypertension |  |  |  |
| No | 551 | 861,573 | 1.00 (reference) |
| Yes | 666 | 243,093 | 2.90 (2.57-3.28) |
| Coronary heart disease |  |  |  |
| No | 1,103 | 1,068,719 | 1.00 (reference) |
| Yes | 114 | 35,946 | 1.28 (1.05-1.57) |
| Stroke |  |  |  |
| No | 1,180 | 1,093,154 | 1.00 (reference) |
| Yes | 37 | 11,511 | 1.13 (0.81-1.57) |

^a^ Hazard ratios (HRs) and 95% confidence intervals (CIs) were generated using Cox proportional hazards models, with adjustment for age at recruitment, gender, dialect, education level, year of interview, body mass index, physical activity, smoking status, alcohol use, total energy intake, total protein intake, red meat consumption, coffee consumption, weekly ginseng intake, weekly medicinal soup intake and self-reported history of physician-diagnosed hypertension, diabetes, coronary heart disease and stroke expect the exposure variables.
